# Dual Active Site in the Endolytic Transglycosylase gp144 of Bacteriophage phiKZ

**Published:** 2017

**Authors:** O.V. Chertkov, G.A. Armeev, I.V. Uporov, S.A. Legotsky, N.N. Sykilinda, A.K. Shaytan, N.L. Klyachko, K.A. Miroshnikov

**Affiliations:** Shemyakin and Ovchinnikov Institute of Bioorganic Chemistry, Mikluho-Maklaya str. 16/10, Moscow, 117997, Russia; Lomonosov Moscow State University, Biology department, Leninskie Gory 1, bld. 12, Moscow, 119991 , Russia; Lomonosov Moscow State University, Chemistry department, Leninskie Gory 1, bld. 11, Moscow, 119991, Russia

**Keywords:** bacteriophage phiKZ, endolysin, enzyme active site, molecular dynamics, site-directed mutagenesis, transglycosylase

## Abstract

Lytic transglycosylases are abundant peptidoglycan lysing enzymes that degrade the heteropolymers of bacterial cell walls in metabolic processes or in the course of a bacteriophage infection. The conventional catalytic mechanism of transglycosylases involves only the Glu or Asp residue. Endolysin gp144 of *Pseudomonas aeruginosa *bacteriophage phiKZ belongs to the family of Gram-negative transglycosylases with a modular composition and *C*-terminal location of the catalytic domain. Glu115 of gp144 performs the predicted role of a catalytic residue. However, replacement of this residue does not completely eliminate the activity of the mutant protein. Site-directed mutagenesis has revealed the participation of Tyr197 in the catalytic mechanism, as well as the presence of a second active site involving Glu178 and Tyr147. The existence of the dual active site was supported by computer modeling and monitoring of the molecular dynamics of the changes in the conformation and surface charge distribution as a consequence of point mutations.

## INTRODUCTION

Bacteriophage phiKZ (vB_PaeM_KZ, GenBank NC_004629) belongs to the *Myoviridae *family and is a type representative of the genus of giant phages that infect the Gram-negative bacteria *Pseudomonas aeruginosa *and some close relatives [1]. PhiKZ-like phages are used as objects in various genomic [2], evolutionary [3], and structural [4-6] studies.

At the late stages of a phage phiKZ infection, the host bacteria are lysed by the peptidoglycan degrading enzyme (endolysin) gp144. This protein has a modular structure and consists of two domains: the *N-*terminal one responsible for primary binding to the substrate, and the *C-*terminal domain with catalytic properties [7]. The peptidoglycan binding function of the *N-*terminal domain of the polypeptide (amino acid residues 9–69) has been confirmed experimentally using a construction with a fused green fluorescent protein [8, 9]. The *C-*terminal domain of gp144 (amino acid residues 70–260) demonstrates strong homology to class 1 lytic transglycosylases. The catalytic mechanism has been proved by mass spectrometric analysis of peptidoglycan cleavage products [10]. Lytic transglycosylases are the enzymes that cleave the β-1,4-glycosidic bond between *N*-acetylmuramic acid (NAM) and *N-*acetylglucosoamine (NAG), yielding the cyclic anhydride of *N*-acetylmuramic acid (the bond between O6 and C1 atoms) [11]. The phiKZ gp144 spatial structures of apoenzyme (3BKV) and the enzyme bound to a chitotetraose molecule (3BKH) were determined by X-ray crystallography with 2.6 Å resolution [12]. The *C-*terminal domain mostly consists of α-helices and is structurally homologous to the catalytic domain of class 1 lytic transglycosylases. According to the widely accepted model [11], the only amino acid residue in the active site is responsible for the catalytic properties: in phiKZ gp144, that residue is Glu115 [12]. However, the mutagenesis of this residue does not completely inactivate the enzyme, leaving ~30% of the activity for the mutant protein [8]. Hence, this study was aimed at shedding light on the role of other amino acid residues in the phiKZ gp144 catalysis and refining the structural organization of the active site.

## EXPERIMENTAL

**Site-directed mutagenesis**

The key manipulations involved in the molecular cloning in *Escherichia coli *were performed as described previously in [13]. Plasmid pKZ144 bearing gene *144 *of bacteriophage phiKZ in a pQE30 (Qiagen) vector was used as the template for point mutagenesis [7]. The QuickChange Kit (Stratagene) was used for site-directed mutagenesis. The following primers were employed for polymerase chain reaction (PCR): E115A Fw 5’-CATTTGCTTCTATT**GCA**TCAGCATTCGATTAC-3’, E115A Re 5’-GTAATCGAATGCTAG**TGC**AATAGAAGCAAATG-3’, H200L Fw 5’-GATCTTTAGCT**CTC**TTCTTTGGGCCTGG-3’, H 2 0 0 L R e 5’-CCAGGCCCAAAGAA**GAG**AGCTAAATAAAGATC-3’, Y197F Fw 5’-ACTGATACTGATCTT**TTT**TTAGCTCACTTCTTT-3’, Y197F Re 5’-AAAGAAGTGAGCTAA**AAA**AAGATCAGTATCAGT-3’. E178A Fw 5’-GCGGAACTGATTAA**GCA**AACATGAACATTCTG-3’, and E178A Re 5’-CAGAATGTTCATGTT**TGC**TTAATCAGTTCCGC-3’.

After PCR, the plasmid carrying the gene encoding the wild-type enzyme was degraded using DpnI endonuclease specific to methylated DNA. The resulting material was used for electroporation of NovaBlue *E. coli *cells (Novagen) plated onto Petri dishes with a LB agar medium containing 100 μg/ml ampicillin, and cultured at 37°C for 16 h. The plasmids pKZ144-E115A, pKZ144-H200L, pKZ144-Y197F, pKZ144-E115A/ H200L, pKZ144-E115A/Y197F, pKZ144-H200L/ Y197F, pKZ144-E178A, and pKZ144-E115A/E178A were isolated from the individual clones using the QiaQuick Spin kit (Qiagen), and sequenced to verify a target mutation.

**Isolation and purification of the proteins**

AD494(DE3) *E.coli *cells (Novagen) transformed with the corresponding plasmids were cultured in a 2xYT medium at 37°C until *A*_600_ ~ 0.6 rel. units; then, expression was induced by adding isopropylthio-β-*D*-galactoside to a final concentration of 0.5 mM. The cells were further incubated at 37°C under moderate aeration for 4 h. Then, the cells from 0.15 L of the culture were precipitated by centrifugation at 3,500 rpm for 15 min; the precipitate was re-suspended in 10 mL of buffer A (20 mM Tris-HCl 8.0, 100 mM NaCl, 1 mM *o-*phenylmethanesulfonyl fluoride (PMSF). The cells were ultrasonically disintegrated (Techpan MD20); the insoluble fragments were separated by centrifugation at 15,000 rpm for 20 min. The supernatant was applied to a Ni-NTA-agarose column (Qiagen). The affine bound protein was eluted with 200 mM imidazole in buffer A and dialysed against 20 mM Tris-HCl pH 8.0, 50 mM NaCl buffer, then stored at –70°C. The protein concentration was determined spectrophotometrically by measuring the absorbance at 280 nm on a GENESYS 10 spectrophotometer (Thermo Electron). The extinction coefficient was calculated using the VectorNTI software based on the concentration of aromatic residues in a protein molecule. The alterations in the secondary structure of mutant proteins were estimated by circular dichroism spectroscopy on a JASCO J-500 spectropolarimeter in a 0.5 cm cell (Hellma) in sodium phosphate buffer, pH 6.2, at room temperature.

**Activity determination**

The enzyme activity of the protein samples was determined using a suspension of cell walls of *P.aeruginosa *PAO1, which were obtained by treating cells with chloroform to remove the outer cell membrane, as a substrate. In order to prepare the cell-wall suspension, PAO1 cells were cultured at 37°C in a 2xTY medium until *A*_500_ ~ 0.6 rel. units was achieved. The cells were precipitated by centrifugation at 4,000 rpm for 15 min. The cell precipitate was re-suspended in a 50 mM Tris- HCl buffer (pH 7.8) saturated with chloroform and incubated under stirring at room temperature for 45 min. The remaining cell walls were precipitated by centrifugation at 4,000 rpm for 15 min. The precipitate was re-suspended in a 10 mM sodium phosphate buffer (pH 6.2, 120 mM NaCl), and absorbance at 500 nm was adjusted to 0.6–1 rel. units. A 30 μl enzyme sample was added to the microplate containing 270 μl of the cell-wall suspension, and the decrease in absorbance was measured on a Victor spectrophotometric reader (Perkin Elmer) at room temperature for 1.5 h with a scan step of 1 min. We assumed that one unit of peptidoglycan lysing activity is the activity that causes a linear decrease in absorbance by 0.001 rel. units per minute. The measurement results were processed in the Activity Calculator software [14]. Three replica experiments were performed; the results were averaged and compared with the corresponding negative controls and the positive control containing hen egg-white lysozyme.

**Molecular dynamics simulations**

The substrate molecule was built and optimized in the Avogadro software [15]. Docking was applied for the relaxed and subsequently frozen native structure using the SwissDock web service [16], and the resulting structure was selected based on an orientation and position similar to the 3BKV PDB structure [12]. The distribution of the electrostatic potential in the native protein molecule was calculated with the PDB2PQR [17, 18] and APBS software [19].

The conformational mobility of the protein was studied with molecular dynamics. The models based on the AMBER99 force field [20, 21] were built according to the crystal structure of 3BKH [12]. Modeling was carried out in the GROMACS software package [22-25] with the following parameters: simulation time, 100 ns; step, 2 fs; the cut-off radius of van der Waals interactions, 1 nm; the electrostatic interactions were taken into account by the PME method; temperature, 300 K; the protein and the solution were thermostated separately; the periodic boundary conditions with the cubic unit cell were used.

The data was analyzed by comparing the root-mean-square deviation of the structures of the mutants from that of the wild-type enzyme. The conformation of transglycosylase obtained by molecular dynamics simulation at 300 K was assumed to be a reference structure. Comparison was performed for the atoms of the protein backbone of only those amino acids that reside near the substrate (amino acids 110–116, 125–150, 168–185, 195–210, 220–230) and can affect its binding to the enzyme. Since the mobility of the molecule at room temperature was too high to draw any statistically valid conclusions about conformational changes at the available calculation times, we used the method of simulated annealing of the structures to lower the temperatures. The system was gradually frozen within 1 ns from 300 K to the temperature of liquid nitrogen (77 K).

## RESULTS

An analysis of the X-ray diffraction data for phiKZ gp144 3BKV [12] demonstrated that the spatial arrangement of Glu115 in the active site with respect to the substrate molecule is not optimal and that there is a possibility that non-canonical amino acid residues are also involved in the catalysis. We suggest that His200 with a reduced electron density, and the highly conserved Tyr197 residue that coordinates a portion of the substrate at the –1 position, could possibly be such residues.

The spatial configuration of phiKZ gp144 3BKV shows that the catalytic domain is very similar to the analogous domain in *E.coli *transglycosylase Slt70 [26]. The position and orientation of the catalytic residue Glu115 in gp144 is similar to the position of Glu478 in Slt70. The mechanism of the catalysis of transglycosylase Slt70 was proposed earlier: it differs from the conventional mechanism by the involvement of an additional Tyr597 residue that is located in the catalytic site of the enzyme and participates in the catalysis. The function of Tyr597 is to activate the catalytic residue Glu478 via the formation of a hydrogen bond, thus increasing the negative charge on Glu478 and facilitating the protonation of the *O-*glycosidic bond [27]. The phiKZ gp144 polypeptide contains a Tyr197 residue with a position and orientation similar to those of Tyr597 in Slt70. The high degree of spatial homology gives grounds for assuming that the mechanism of catalysis of phiKZ gp144 is similar to that of Slt70.

**Fig. 1:**
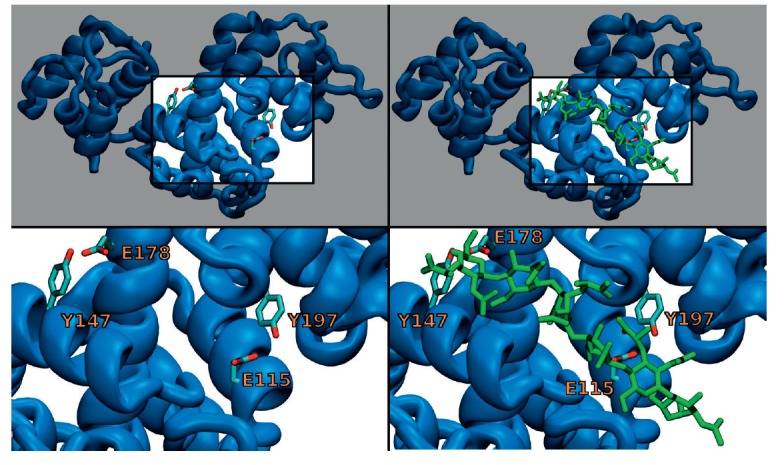
The side chains of amino acid residues forming the active sites of phiKZ gp144 and the possible location of the NAM–NAG molecule. The top image shows the general view of the enzyme without (top left) and with the substrate (top right). Bottom images provide a close-up view of the binding sites of the enzyme without (bottom left) and with the substrate (bottom right).

The key hypothesis explaining why enzyme activity is retained after the main catalytic residue is replaced suggests that there exist two catalytic sites: the principal one, Glu115/Tyr197, and a secondary active site, Glu178/Tyr147. The arrangement of amino acids in the putative secondary active site with respect to the chain of the peptidoglycan substrate is similar to that in the principal active site. Superposition of the side chains of the residues of the principal and secondary active sites by calculating the minimal root-mean-square deviation yields an almost identical position (*Fig. 1*).

A series of single and double point mutants of these amino acids was obtained to experimentally confirm the role of the residues partaking in the functioning of the active site of phiKZ gp144. Insertion of point mutation had no significant effect on the solubility or the secondary structure (as shown by the binding to the affinity column and circular dichroism spectroscopy). The mutant proteins were purified by Ni-chelate chromatography [7] without any significant modifications. The resulting specimens contained 3–6 mg of the mutant protein with > 90% purity.

**Fig. 2:**
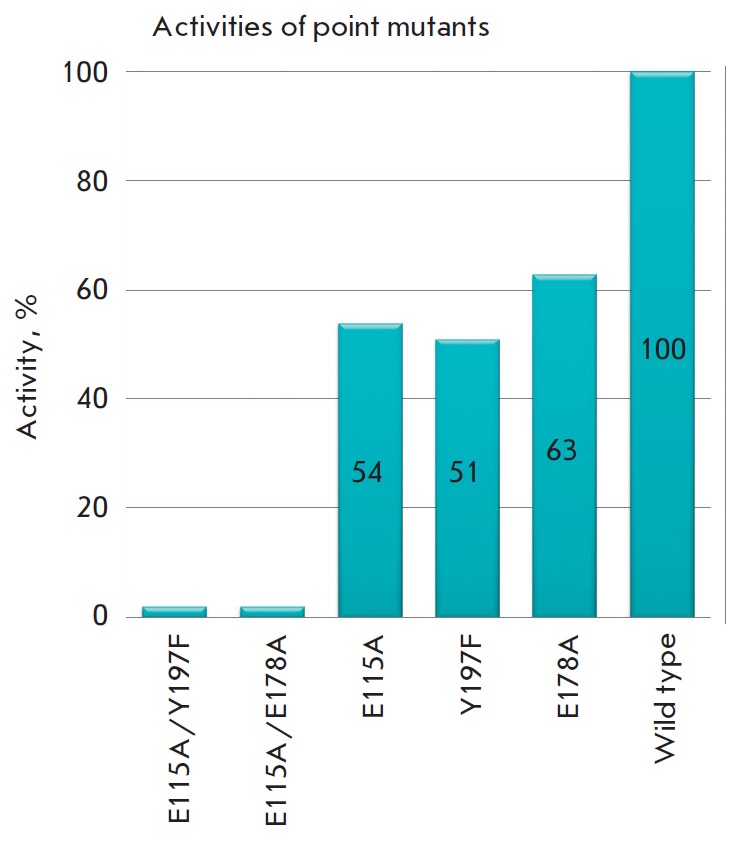
Activities of single- and double-point phiKZ gp144 mutants compared to that of the wild-type enzyme taken as 100%.

The single point mutants E115A, E178A, and Y197F (with respect to each individual residue) had residual activities of 54, 63, and 51%, respectively, compared to the intact phiKZ gp144 protein (*Fig. 2*, *Table 1*). All the mutants exhibited maximum activity in a buffer with the same composition (pH 6.2 and *I *= 120 mM NaCl) as that for the wild-type enzyme. All subsequent reactions proceeded under the same conditions. Mutation in His reduced activity by 20–30%, thus supporting the hypothesis that histidine residues are involved in substrate coordination.

**Table 1:** Activities of single- and double-point mutants phiKZ gp144, and the wild-type enzyme.

Enzyme	Wild type	E178A	Y197F	E115A	E115A/ Y197F	E115A /E178A
Activity, U/mg	210000	132000	107000	113000	0	0

The fact that E115A/Y197F with mutations in both residues of the principal active site and the intact secondary site lost its activity lacks consistency under this hypothesis. In order to interpret this phenomenon, we had to study both the details of the spatial interplay between the protein and the substrate and the possible changes in protein conformation caused by mutations. Since the conformation of the chitotetraose used to emulate the substrate in phiKZ experiments on crystallization of gp144 [12] differs rather significantly from that of the natural substrate (NAM-NAG)3, molecular docking was carried out to compute the possible configurations of substrate binding to the protein molecule. The most plausible arrangement of the substrate in the active site groove is shown in *Fig. 1*.

**Fig. 3:**
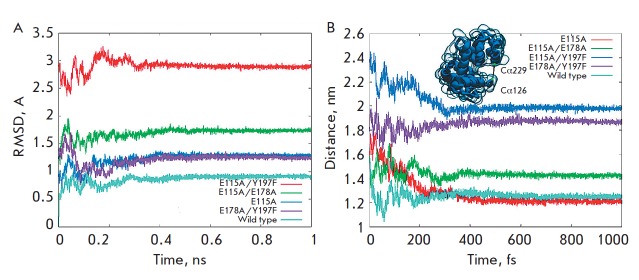
A – The root-mean-square deviation of the protein backbone of transglycosylases frozen at 77 K. The deviation was calculated only for the area in contact with the substrate. B – Changes in spacing between the Cα atoms of the amino acids 126 and 229 during freezing up to 77K.

**Fig. 4:**
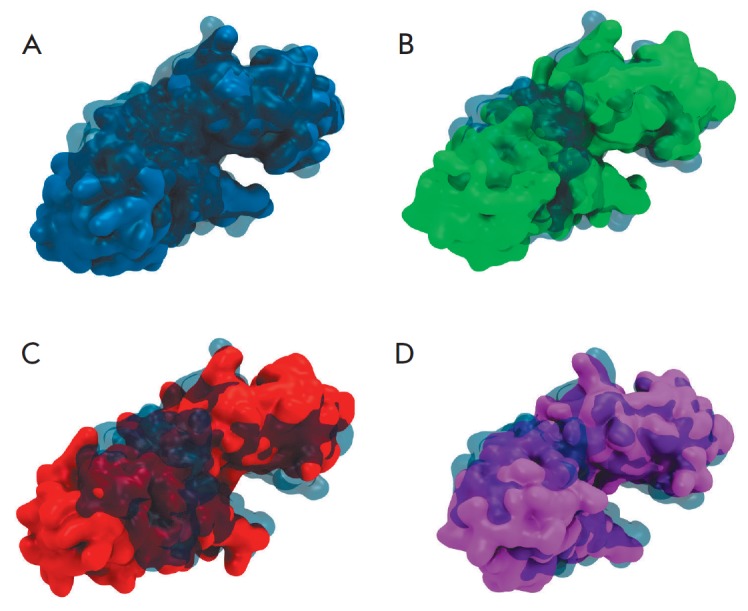
Surface modeling of the phiKZ gp144 mutants compared to the native form: A – E115A, B – E115A/ E178A, C – E115A/Y197F, and D – E178A/Y197F. The translucent surface belongs to the structure of the wild-type enzyme. The domain not involved in binding to the substrate is deleted in the images. The side view of the groove is provided.

In order to additionally evaluate the changes in protein conformations with various substitutions, we carried out a molecular dynamic analysis of the groove configuration by studying its appearance (*Fig. 3A*) and the distance between the Cα atoms of the amino acid residues 126 and 229 (*Fig. 3B*, *Table 2*). It is most likely that some mutations disrupt the interaction network between the side chains of the residues forming the active site; so, the domain of the active side groove of phiKZ gp144 changes its conformation rather significantly. For example, mutations in amino acid residues in the principal active site change the shape of the groove where the substrate is packaged, which may reduce protein affinity to the substrate and, therefore, its reactivity. One can see that a double mutation in the principal active site results in the most significant groove opening (*Fig. 4*).

**Table 2:** Standard deviations of the protein structure during simulated annealing to the temperature of liquid nitrogen.

Enzyme	Mean, Å	Standard deviation, Å	Min, Å	Max, Å
Native	0.858	0.081	0.005	0.974
E115A	1.225	0.118	0.692	1.370
E115A /Y197F	2.895	0.104	2.366	3.249
E178A /Y197F	1.219	0.110	0.762	1.693
E115A /E178A	1.693	0.084	1.130	1.944

**Fig. 5:**
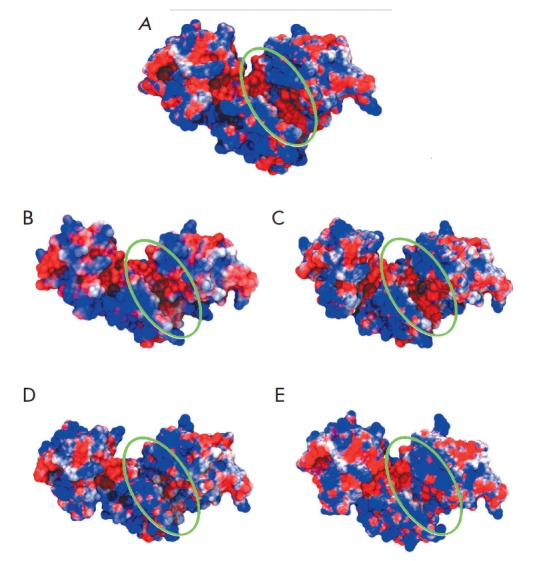
Charge distribution on the surface of the mutant phiKZ gp144 isoforms. The substrate-binding site is shown with a circle. A) native structure, B) E115A, C) E178A/ Y197F, D) E115A/E178A, and E) E115A Y197F.

Charge distribution over the surface of each mutant accessible to a solvent was calculated. The calculation of the surface charge of a phiKZ gp144 protein globule shows that the substrate-binding groove has a predominantly positive charge. Substitutions of amino acid residues in active sites also significantly change the charge distribution in a number of cases (*Fig. 5*). The E115A/Y197F mutant exhibited no activity, although one of the active sites remained intact. The double point mutation in the closely spaced amino acids drastically changed the conformation of the substrate-binding surface (*Fig. 4*) and altered the charge in this portion of the groove (*Fig. 5*). This change in surface properties has the potential to hinder substrate binding by this phiKZ gp144 mutant, thus completely inactivating the enzyme.

## DISCUSSION

Lytic transglycosylases belong to the class of peptidoglycan lysing enzymes that play a crucial role in the life cycle of bacteria [28] and bacteriophages [29]. Transglycosylases affect the same peptidoglycan domain as lysozymes ([EC 3.2.1.17]; peptidoglycan-N-acetylmuramoylhydrolases, muramidases): the β-1,4-glycosidic bond between the NAM and NAG residues. The key difference between transglycosylases and muramidases is that transglycosylases contain no nucleophilic catalytic residue. The differences are also manifested in the binding of the oligosaccharide at the binding sites +1 and +2. The classical mechanism of action of transglycosylases suggests that there is one acidic catalytic residue that resides in the active site of the enzyme between the subsites +1 and –1. At the first stage of the reaction, the catalytic amino acid residue protonates the glycosidic oxygen, resulting in the formation of an oxocarbonium cation and further rupture of the *O-*glycosidic bond between NAM and NAG. The second stage of the reaction involves the intramolecular nucleophilic attack of carbon C1 of the oxocarbonium cation by C6 hydroxyl, yielding the 1,6-oxazalone cycle of NAM. The catalytic residue Glu activates C6 hydroxyl by pulling a proton away from the hydroxyl [11].

**Fig. 6:**
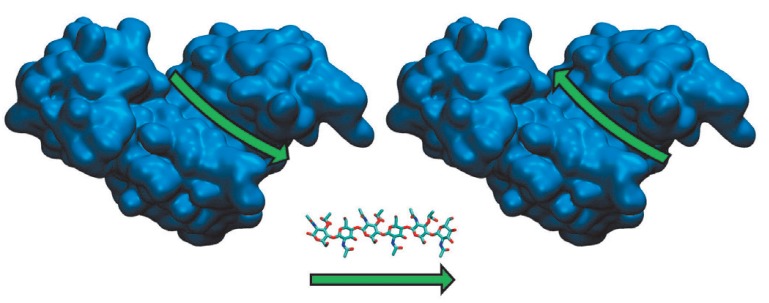
The possible directions of substrate orientation in the groove of phiKZ gp144. The substrate (*N-*acetylmuramyl-*N-*acetylglucosamine) is shown above the arrow.

We suggest that there are two active sites in the polypeptide chain of phiKZ gp144 transglycosylase : a principal one (E115/Y197) and a secondary (E178/ Y147) one. A portion of a peptidoglycan molecule can be bound to the groove on the enzyme globule in two ways (*Fig. 6*). The direction of substrate packaging probably matters for the reaction and determines which active site will be responsible for the reaction of substrate degradation. Hence, the corresponding direction of substrate packaging will not be accompanied by enzyme-induced cleavage if one of the domains of the active site is inactivated.

Glu is the key residue that attacks the β-1,4- glycosidic bond. Its substitution results in complete inactivation of one of the active sites, thus reducing enzyme activity twofold. Single-point mutations at the Tyr residue also reduce enzyme activity. In the spatial model of the active site, Glu and Tyr face one another and form a hydrogen bond. They are coordinated in this fashion during almost the entire molecular dynamics simulation. One can suggest that Tyr immobilizes Glu in the spatial position most favorable for the reaction. The substitution of Tyr for Phe shifts the side chain of Glu away from the favorable position and changes the activity of the mutant site. No site activation was detected in the experiment with the double mutant E178A/Y197F. Although one of the active sites was switched off because of Glu substitution, the second one remained active even while lacking the Tyr residue. The double mutant E115A/E178A exhibited no activity, since the attacking Glu residues in both active sites had been substituted.

The mutant E115A/Y197F exhibits no activity despite the fact that one of the active sites remained in its native state. Double mutation in closely spaced amino acids deeply altered the conformation of the substrate-binding surface (*Fig. 4*) and the charge in this groove portion (*Fig. 5*). This alteration of surface properties probably prevents phiKZ gp144 from binding to the substrate; so, the enzyme becomes completely inactive.

## CONCLUSIONS

Our findings support the hypothesis that phiKZ gp144 possesses two active sites and elucidate the catalytic mechanism of this enzyme. The dual active site probably appeared in the course of evolution, as indicated by the high homology between the active site structures.

Numerous strains of pathogenic and opportunistic bacteria resistant to synthetic antibiotics are currently emerging. Enzybiotics, enzymes toxic to bacteria (e.g., peptidoglycan hydrolases), can potentially be used as an alternative to antibiotics [29]. One of the most efficient approaches to designing enzybiotics that would be active with respect to Gram-negative pathogens involves combining enzymes and polycationic peptides [30] or constructing fusion proteins that comprise such peptides (artilysins) [31, 32]. Polycationic peptides fa cilitate enzyme penetration through the bacterial outer membrane. In this context, the catalytic domain of phiKZ gp144 is a suitable candidate for designing engineered enzymatic antimicrobial drugs due to its high activity and specificity. A thorough understanding of the mechanisms of action of peptidoglycan lysing enzymes would enable us to use them to design more effective agents for combating pathogenic bacteria.

## Abbreviations

gp: gene product
NAM: N-acetylmuramic acid
NAG: N-acetylglucosoamine
